# The ninth ENBDC Weggis meeting: growth and in-depth characterisation of normal and neoplastic breast cells

**DOI:** 10.1186/s13058-017-0891-9

**Published:** 2017-08-22

**Authors:** Katrin E. Wiese, Romain J. Amante, Maria dM. Vivanco, Mohamed Bentires-Alj, Richard D. Iggo

**Affiliations:** 1 Section of Molecular Cytology and Van Leeuwenhoek Centre for Advanced Microscopy, Swammerdam Institute for Life Sciences, University of Amsterdam, 1098 XH Amsterdam, The Netherlands; 2 Department of Biomedicine, University of Basel, University Hospital Basel, Hebelstrasse 20, CH-4031 Basel, Switzerland; 3 CIC bioGUNE, Technological Park Bizkaia, 48160 Derio, Spain; 4 INSERM U1218, Institut Bergonie, University of Bordeaux, 229 cours de l’Argonne, 33076 Bordeaux, France

**Keywords:** Breast cancer, Oestrogen receptor, Proteomics, Organoid culture, CRISPR screen, European Network for Breast Development and Cancer

## Abstract

Mammary gland biologists gathered for the ninth annual workshop of the European Network for Breast Development and Cancer (ENBDC) at Weggis on the shores of Lake Lucerne in March 2017. The main themes were oestrogen receptor alpha signalling, new techniques for mammary cell culture, CRISPR screening and proteogenomics.

## Main text

The workshop started with a keynote lecture by *Jason Carroll* (CRUK, Cambridge, UK), who shed new light on the relationship between oestrogen and progesterone receptors in breast cancer. ChIP-sequencing studies revealed that activated progesterone receptor (PR) redirects oestrogen receptor alpha (ER) binding to genomic sites that are associated with better survival of patients, leading Carroll to propose that *PGR* is a tumour suppressor gene [1]. To test this idea, Carroll has initiated a window trial of megestrol to activate PR in ER+ breast cancer. Putting hormone sensing (sensor) cells in a broader context, *Tan Ince* (University of Miami, USA) reviewed his classification of normal human breast cells according to ER, androgen receptor (AR) and vitamin D receptor (VDR) expression [2], explaining the logic of phylogenetic and cladistic classification strategies and how to select the best cell-type specific markers (those with bimodal expression).

Preserving ER expression during culture is one of the main challenges in the field. Last year we learnt that the mammary ducts create a special microenvironment that supports the growth of ER+ human breast cancer [3, 4]; this year we learnt how to reproduce this microenvironment in culture. *Lone Ronnov-Jessen* (University of Copenhagen, Denmark) described how her laboratory first identified new cell surface markers for ER+ cells, and then demonstrated that inhibiting TGFβ receptor signalling helps to sustain ER expression in cells expressing those markers [5]. Taking a similar approach to isolating subpopulations of fibroblasts, she showed that normal breast epithelial cells form tubules if plated on a feeder layer of intralobular fibroblasts. *Oded Kopper* (Hubrecht Institute, Utrecht, the Netherlands) presented work from Hans Clevers’ laboratory on organoid cultures of normal mammary epithelial cells and breast cancer. The organoid approach starts from the assumption that the best way to grow tumour cells is to mimic physiological conditions. The Clevers laboratory has pioneered the organoid culture technique where cells are embedded in matrigel, allowing them to grow in a self-organising manner in 3D [6]. This format makes it easy to test multiple different growth factors and inhibitors. With a cocktail of growth factors (EGF, R-Spondin 1, FGF7/10 and NRG1) and inhibitors (for BMP, TGFβ, ROCK and p38) that presumably mimics the paracrine environment (“niche”), they were able to establish 101 breast cancer organoids from 151 tumours tested, including all of the major types of breast cancer. A different organoid approach based on technology from OcellO, a Dutch company that performs drug screening in 3D organoid cultures, is already finding a place in clinical studies, as described by *Rebecca Marlow* from Andrew Tutt’s laboratory (King’s College, London, UK). Although very promising, some aspects of the technology, such as the composition of the hydrogels, are proprietary, which means the Clevers system is likely to sweep the field, at least among basic scientists.

Traditionally, Weggis always features an “off-the-wall” talk about new technologies from someone outside the field. This year it fell to *Pierre Nassoy* (University of Bordeaux, France) to describe his alginate microencapsulation technology [7]. The capsules enclose cells in a thin skin of alginate that provides physical constraint. It is possible to generate thousands of capsules in a few minutes, making it possible to study huge numbers of individual clones. The technology can also be used with mixtures of different cell types to mimic the interactions between, for example, tumour cells and cancer-associated fibroblasts. The size and thickness of the capsules can be modified to change the physical properties of the microenvironment. In a final flourish, Nassoy showed that it is even possible to generate long tubes of alginate that could perhaps be used to encapsulate entire mammary ducts. *Barbara Szczerba* from the Aceto laboratory (University of Basel, Switzerland) then described a microfluidic-based technology to isolate and grow clusters of circulating tumour cells from blood samples [8, 9]. The culture system is similar to classic mammosphere culture but preserves ER expression in a substantial proportion of cases, perhaps because the cells are already adapted to surviving in suspension.

*Alexandra Van Keymeulen* from the Blanpain group (Free University of Brussels, Belgium) first addressed the controversy surrounding her landmark 2011 *Nature* paper proposing that the adult gland contains distinct long-lived unipotent luminal and basal stem cells [10]. That work was challenged by a paper from the Visvader laboratory arguing that bipotent stem cells play a major role in normal homeostasis of the adult gland [11]. To resolve this issue, Van Keymeulen has now performed experiments with the Confetti mouse model that support her original conclusions [10]. Arguably, the main interest of the work is the confirmation that long-lived unipotent stem cells exist. Van Keymeulen ended the ER talks on a high note by performing lineage tracing with Esr1-rtTA/tetO-cre/Rosa-lsl-YFP mice. She showed that long-lived unipotent ER+ stem cells exist in the adult gland. They are prime candidates for the cell of origin of classic ER+ human breast cancers. Previous work from the Blanpain and Bentires-Alj laboratories showed that PIK3CA mutations break the lineage restriction of luminal and basal progenitors [12, 13]. It will be fascinating to see whether the same is true of the new ER+ stem cell population.

For aficionados of CRISPR technology, there was a group of talks on pooled library screens. *Reuven Agami* (NKI, Amsterdam, the Netherlands) described genetic screens to identify functional elements in non-coding DNA [14]. He characterised enhancers targeted by p53 and ER, and found sites important not only for binding by the primary factors but also for cooperating transcription factors. This is an extremely elegant approach but the technology is still a long way from being able to serve genome-wide studies. *Björn von Eyss* (Leibniz Institute on Aging, Jena, Germany), winner of the DeOme prize 2017 for best short talk, then described a CRISPR screen for MST/LATS-independent regulators of YAP/TAZ in a human breast cell line. *Ilirjana Bajrami*, from Chris Lord’s laboratory (The Institute of Cancer Research, London, UK), then described siRNA screens looking for genes showing synthetic lethality with CRISPR-engineered loss of E-cadherin. The upshot is that loss of ROS1 causes a mild cytokinesis defect that is made far worse by loss of E-cadherin. Drugs like crizotinib that inhibit ROS1 kill the lobular tumour cells in Jos Jonkers’ *Cdh1*-null mouse models [15]. A phase II trial is now under way to see whether humans are as obedient as mice.

Novel techniques to better understand the breast cancer proteome and their potential to improve cancer therapy were the focus of the Proteomics Session (chaired by Romain Amante, University of Basel, Switzerland and Katrin Wiese, University of Amsterdam, the Netherlands). *Johanna Wagner* from Bernd Bodenmiller’s laboratory (University of Zürich, Switzerland) discussed how proteomics can reveal breast cancer heterogeneity at the single cell level. Using metal-tag barcoding, they simultaneously profiled 38 markers in tumour samples. This information could potentially form the basis for individualised treatment strategies. Next, *Janne Lehtiö* (Karolinska Institute, Stockholm, Sweden) introduced the emerging field of proteogenomics and highlighted the importance of adding proteome information as an additional data layer in cancer studies. The quantitative methods his laboratory is developing can aid biomarker discovery and identify protein signatures associated with drug resistance [16, 17]. Importantly, the data will soon be shared with other researchers in the form of a database. *Simone Lemeer* (Utrecht University, the Netherlands) gave a thorough introduction about the principles of mass spectrometry, and then presented a study that combined different proteomic and metabolic techniques to identify the mechanism of lapatinib resistance in breast cancer [18]. Finally, *Jukka Westermarck* (Centre for Biotechnology, Turku, Finland) stressed the significance of inhibition of PP2A-mediated protein de-phosphorylation for malignant transformation, and discussed PP2A inhibitor protein CIP2A as a promising target for breast cancer therapy.

Two talks described genomic characterisation of tumours to understand specific phenotypes. *Leonie Young* (RCSI, Dublin, Ireland) described studies trying to explain why particular subtypes of breast cancer metastasise to particular sites. *Therese Sorlie* (Oslo University Hospital, Norway) described a set of tumours induced in mice by exposure to MPA and DMBA. Use of DMBA led to a 7-fold higher mutation rate than is seen in human tumours, but the most common changes included mouse homologues of many known human breast cancer genes, like *TP53*, *NF1*, *ATR*, *KRAS* and *KMT2C*.

The meeting closed with a presentation on chemotherapy-induced tumour dormancy mediated by IRF7-dependent activation of interferon signalling (*Sanam Peyvandi*, Ruegg laboratory, University of Fribourg, Switzerland), and a beautiful imaging study of proliferation in explant cultures of mammary buds from FUCCI mice (*Riitta Lindstrom*, Mikkola laboratory, University of Helsinki, Finland).

## Conclusions

This was the year when culture of all subtypes of breast cancer came of age. It will be interesting to see how quickly the ability to study tumours from individual patients moves into clinical practice. Currently, the main barrier to using NGS data from patients is the inability to predict who will respond to genomically guided therapy. Rapid functional testing in the new culture systems presented at the meeting would go a long way towards solving this problem.

The 10th ENBDC meeting is set for 15–17 March 2018 and the meeting will be chaired by Eva Gonzalez Suarez (Bellvitge Institute for Biomedical Research, Barcelona, Spain).

## Abbreviations

AR: Androgen receptor
ENBDC: European Network for Breast Development and Cancer
ER: Oestrogen receptor alpha
PR: Progesterone receptor
VDR: Vitamin D receptor
